# Mapping Metabolite and ICD-10 Associations

**DOI:** 10.3390/metabo10050196

**Published:** 2020-05-14

**Authors:** Egon Taalberg, Kalle Kilk

**Affiliations:** 1 Department of Biochemistry, Institute of Biomedicine and Translational Medicine, University of Tartu, 50411 Tartu, Estonia; egon.taalberg@ut.ee; 2 Centre of Excellence for Genomics and Translational Medicine, 19 Ravila Street, 50411 Tartu, Estonia

**Keywords:** metabolomics, ICD-10, AUC-ROC, specificity, sensitivity, biomarker, comorbidity

## Abstract

The search for novel metabolic biomarkers is intense but has had limited practical outcomes for medicine. Part of the problem is that we lack knowledge of how different comorbidities influence biomarkers’ performance. In this study, 49 metabolites were measured by targeted LC/MS protocols in the serum of 1011 volunteers. Their performance as potential biomarkers was evaluated by the area under the curve of receiver operator characteristics (AUC-ROC) for 105 diagnosis codes or code groups from the 10th revision of the international classification of diseases (ICD-10). Additionally, the interferences between diagnosis codes were investigated. The highest AUC-ROC values for individual metabolites and ICD-10 code combinations reached a moderate (0.7) range. Most metabolites that were found to be potential markers remained so independently of the control group composition or comorbidities. The precise value of the AUC-ROC, however, could vary depending on the comorbidities. Moreover, networks of metabolite and disease associations were built in order to map diseases, which may interfere with metabolic biomarker research on other diseases.

## 1. Introduction

Various technological advantages and, in particular, different “-omics” studies have led to an increased number of associations between diseases and genes or biomolecules. Despite the extensive reporting of novel potential biomarkers for early disease detection or monitoring, they rarely make it into clinical practice [1,2,3]. The first reason is that the validation of a biomarker for general use requires greater effort and resources than its determination in a small study group, which means that many potential markers are not studied further. The second reason for failures is the heterogeneity of the general population, which makes the statistical significances of small, specifically designed studies fade away. The latter effect may of course be due to the reported potential biomarker being a false positive hit in the original study, but it may also be due to the inability to account for comorbidities and other influencing factors in the validation study. Without comprehensive information on the possible influencing factors, one cannot determine a biomarker’s suitability for a personalized medicine approach.

Personalized medicine or “precision medicine” can be understood as not having strict treatment guidelines, but instead involving the selection of treatment based on the molecular background of the patient and the disease. The term is most frequently associated with genetics and possibly the most illustrative success is in the field of oncology [4]. However, one should keep in mind that although genetic data determine an algorithm for organism development and strengths and weaknesses, the real outcome is more properly displayed at the protein and metabolite levels, which depend on both genetic and environmental factors and form the bulk of organic life. Therefore, despite challenging technical issues, proteomics and metabolomics have gained attention and proposed many novel protein or metabolite markers for various diseases [5,6,7,8,9].

Many metabolites (incl. amino acids, phospholipids and simple organic acids) have been reported as potential biomarkers. In relation to this study, the most relevant examples are [10,11,12,13,14,15,16,17], but the list is longer and continuously growing. While thorough metabolomic analyses have been conducted for specific diseases, the opposite—systematically determining diseases associated with a specific metabolite—has not been conducted. The main classification of diseases used today by healthcare systems and researchers around the world is still the 10th revision of the international classification of diseases (ICD-10). ICD-11 is anticipated, but the metabolomic input for any further update requires mapping metabolite and disease classification relations.

The systematic mapping of metabolite and disease associations is important for designing and interpreting studies designed for biomarker research. Furthermore, the specificity and sensitivity of potential metabolic biomarkers have gained relatively little attention, although they are key parameters for translating markers into clinical use [18]. When characterizing a biomarker, its specificity and sensitivity depend on a threshold value, and by changing it, we can increase the sensitivity by reducing the specificity or vice versa. Therefore, the area under the curve of receiver operator characteristics (AUC-ROC) or concordance statistics are more commonly used in biomarker performance comparisons. The curve of receiver operator characteristics shows the dependence of true and false positive rates, and the area under this curve is essentially the probability of a correct diagnosis. An AUC-ROC value of 0.5 means that the marker for which the AUC-ROC is calculated is as reliable as a coinflip, and an AUC-ROC value of 1 means that the marker never misdiagnoses.

The present study aimed to evaluate how the AUC-ROC values of potential metabolic biomarkers depend on comorbidities and the choice of control group. In addition, networks of disease and metabolite relationships were built to visualize not only metabolite and disease associations, but also the synergistic and antagonistic relations of diseases at the metabolite level.

## 2. Results

### 2.1. Characterization of the Study Group

**Figure 1:**
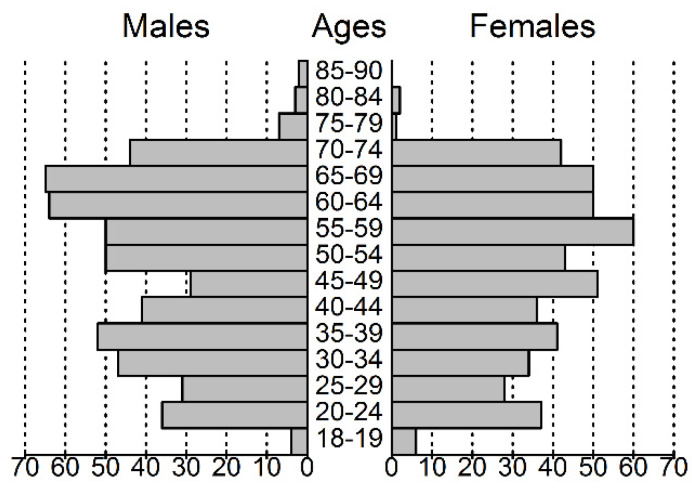
Population pyramid of the volunteers participating in this study.

Of the 1011 enrolled individuals, 526 (52%) were male, and compared to the general population older age groups were overrepresented (Figure 1). All the participants reported at least one medical problem that could be associated with an ICD-10 [19] diagnostic code (incl. O-Z codes). After the removal of diagnoses of F (somatoform or anxiety) and O-Z codes, as well as myopia, dental caries and dental anomalies, 36 (M/F = 26/10) patients with a mean age of 36.1 (23–68) years were found to have no diagnoses. This group is referred to as “healthy controls”. In total, 105 ICD-10 categories (excluding categories differing in specification details) remained, 65 of which were found in more than 50 cases. The most frequent diagnoses or diagnosis groups with their age and gender proportions are shown in Table 1.

### 2.2. Selection of Metabolic Biomarker Candidates

The Pubmed database was screened for original and review articles for metabolomic biomarkers of diabetes, obesity, cardiovascular diseases and chronic pulmonary diseases in 2016–2017. Furthermore, it was tested with respect to whether the proposed markers could be reliably detected and quantified in our lab. The final selection consisted of alanine (Ala); arginine (Arg); glycine (Gly); glutamine (Glu); methionine (Met); leucine and isoleucine (Leu + Ile; summary concentration measured due to technical reasons); valine (Val); phenylalanine (Phe); tyrosine (Tyr); tryptophan (Trp); ornithine (Orn); symmetric and asymmetric dimethyarginines (SDMA and ADMA); carnitine esters with 2, 3, 4, 5, 16 and 18 carbons long fatty acid residues; free carnitine (C2, C3, C4, C5, C16, C18 and C0, respectively); 3-hydroxybutyrate; 2-hydroxybutyrate; arachidonic acid; eicosapentaneoic acid; hexoses; lactate; creatine; creatinine; dimethylglycine (DiMeGly); betaine; kynurenine; kynurenic acid; taurine; oxoproline; homocysteine (HCys); uric acid; and nine different phospholipid species—PC(C32:1), PC(C36:1), PC(C36:3), PC(C38:6), PC(C40:5), lysoPC(C16:0), lysoPC(C18:0), lysoPC(C18:2) and lysoPC(C22:6) [10,11,12,13,14,15,16,17,20,21,22,23].

### 2.3. Receiver Operator Curves for Individual Disease Categories

**Figure 2:**
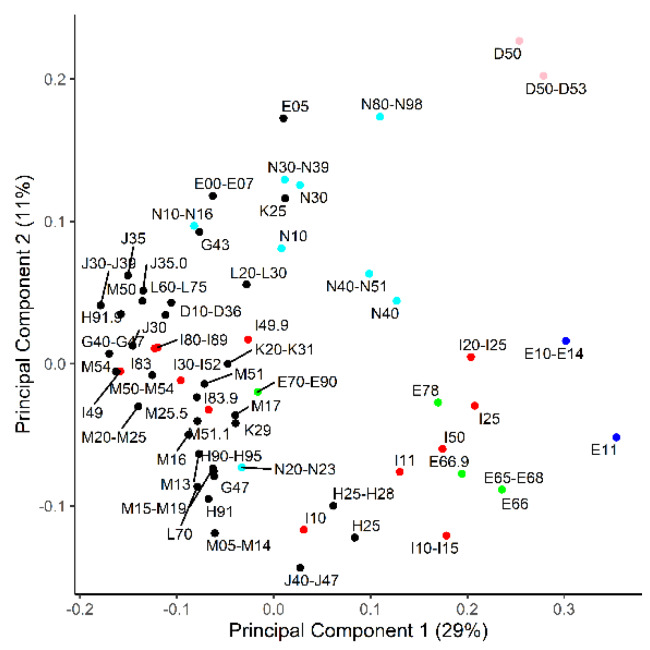
Principal component analysis on how ICD-10 codes relate to each other based on area under the curve of receiver operator characteristics (AUC-ROC) values from a set of 49 metabolites. Components 1 and 2 are the shown on the x- and y axes, respectively, and explain the denoted percentage of total variance. Red—diseases of the circulatory system; blue—diabetes; green—obesity and dyslipidemia; pink—anemia; cyan—diseases of the genitourinary system.

The AUC-ROC values and binomial logistic regression were calculated for each individual metabolic biomarker for each disease category or category group (cases > 50). The highest values are presented in Table 2, and all the values are presented in Table S1. The average AUC-ROC over all the calculations was 0.54 ± 0.05. To better visualize the diseases that may share metabolic biomarkers, a principal component analysis (PCA) on the AUC-ROC matrix was performed (Figure 2). The first principal component was most dependent on branched-chain amino acids (BCAA), Ala, Glu, C3-carnitine, DiMeGly, kynurenine, urea and separated anemia (D50–D53), obesity (E66), diabetes (E10–E14), coronary ischemia (I20–I25) and hypertension (I10–I15) from the bulk of the diagnosis categories. The second component had the highest loadings for Leu + Ile, lysoPC(C22:6), even chain-length acylcarnitines, 2-hydroxybutyrate, hexoses and uric acid. The second component had anemias (D50–D53) at one end and chronic lung diseases (J40–J47; asthma and chronic obstructive lung disease mainly) at the other.

**Figure 3:**
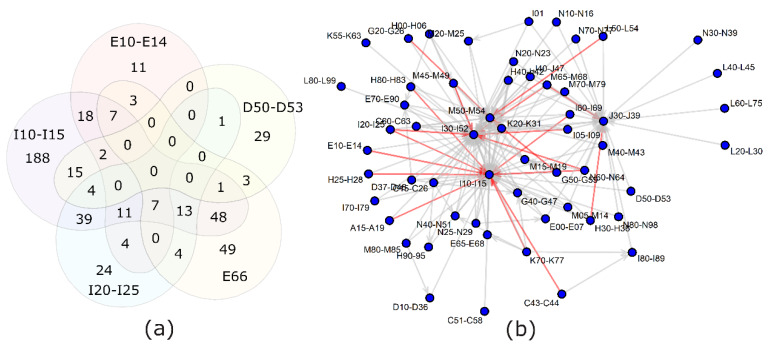
Overlap between diagnosis groups. (**a**) Relations between the 5 diagnosis groups separated from the bulk of diagnoses by principal component analysis: E66—obesity; D50–D53—anemia; E10–E14—diabetes; I10–I15—hypertension; I20–I25—ischemic heart diseases. (**b**) A more comprehensive view of the comorbidities among the study population. Gray arrows—40–60% of people with the diagnosis at the beginning of the arrow have also the diagnosis at the end of the arrow. Red arrows—over 60% overlap for the diagnoses.

In order to exclude the possibility that diseases display identical markers as a consequence of always being co-present, overlaps among the disease-reporting subpopulations were calculated (Figure 3). The results demonstrate that most ICD-10 codes have a fraction of patients without comorbidities and another fraction with one or more comorbidities.

**Figure 4:**
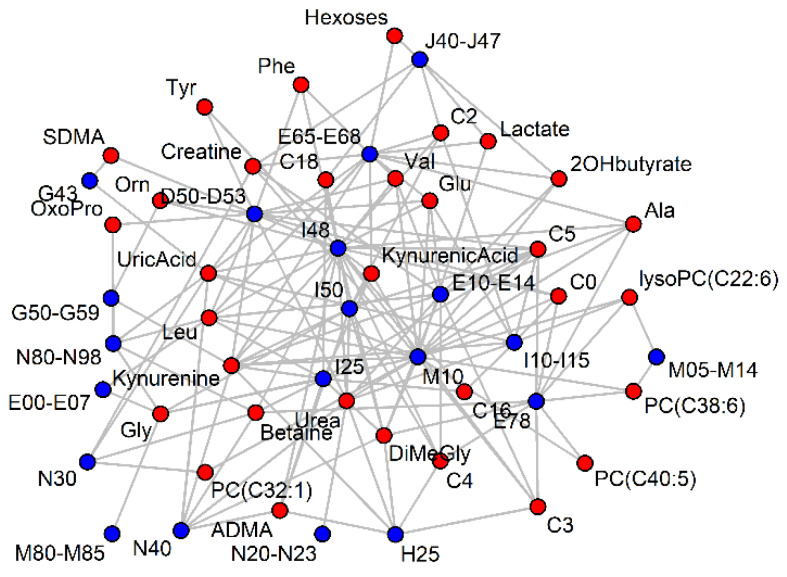
Significant (*p* < 0.001) AUC-ROC values (> 0.6) of serum metabolites (red) for ICD-10 codes (blue). For clarity, most ICD-10 codes have been grouped and vertices with less than two connections have been removed.

A network of metabolites and diseases was built to visualize the diseases that share metabolic markers (Figure 4). A similar network was built based on significant beta values in logistic regression (Figure not shown due to a high similarity to Figure 4).

### 2.4. Importance of Healthy Controls

The control group in biomarker discoveries is most often composed of healthy persons or persons with no related or chronic diseases. Recruiting perfectly healthy controls is often not possible in general practice, and the decision as to which other diseases interfere and should be excluded is arbitrary. Therefore, we also calculated the AUC-ROC values in comparison to the healthy subgroup, instead of the general population.

Comparing to the completely healthy subset, instead of the general population, frequently increased the AUC-ROC, and the average was 0.58 ± 0.07 (Table S1). If, with the general population, 1664 marker and ICD-10 combinations had an AUC-ROC of significantly above 0.5, then by using the healthy control group this count rose to 2194. Leaving aside the 0.5 threshold and performing a direct statistical comparison of the AUC-ROC values revealed, however, much less significant differences between the two control groups. A comparison against healthy controls yielded 158 new potential markers and a loss of 4 markers. The composition of the control group affected about 14% of marker-disease combinations, when also including cases where a previously significant value became much more significant.

Hydrophobic metabolites (phospholipids, long chain carnitine esters, arachidonic acid) were more frequently sensitive to the control group than hydrophilic metabolites or specific diagnosis codes. This implies a difference in lipid metabolism between the general population and very healthy individuals.

### 2.5. The Case of an Underlying Disease

The interactions of comorbidities were analyzed in more detail. First, combinations of any disease within the population of any another disease were studied—for example, coronary artery disease in people with hypertension. The criteria were that there were at least 15 cases with both diagnoses, 50 cases of the underlying comorbidity and at least 15 of those without the primary disease. With these values, an AUC-ROC of 0.8 would have a power of 0.95 at the 95% confidence level. In total, 2206 combinations fitted these criteria.

In 831 unique combinations, the resulting AUC-ROC was significantly different from its values for either individual diagnoses (Table 3 and Table S2). In 78 cases, a metabolite with an AUC-ROC significantly above 0.5 for both diagnoses became insignificant when the diagnoses existed as comorbidities. This is expected to happen if the disease of interest and underlying disease affect the metabolite independently and roughly equally, with no synergy or antagonism.

In 458 cases, a metabolite appeared to be significant if two diagnoses were both present, although the same metabolite was an insignificant marker for either diagnosis separately. The top two AUC-ROC values > 0.8 were if migraine, headache or sleep apnea syndromes (G40–G47) co-existed with either anemia (D50–D53) or a history of acute myocarditis (I40) (see also Table 3).

### 2.6. Combination of Two Diseases

Next, people with two disease combinations were compared to the general population. For example, people with both hypertension and coronary artery disease in comparison to everyone else, including people with only one of the mentioned diagnoses.

One hundred and twelve unique combinations were found where a metabolite was shown to have a significantly (*p* < 0.05 with a power of >0.95) different AUC-ROC value for a two-disease combination compared with both diseases analyzed separately (Table 4, Table S3). In 100 combinations, at least one disease was from endocrine/nutritional (E codes) or cardiovascular diagnoses (I codes). More specifically, obesity (E66) and hypertension (I10–I15) were the most frequent diagnoses in this list, which may partly stem from the fact that the marker selection for the study was based on previous reports on cardiovascular, diabetic and chronic respiratory diseases. Both the E and I codes had a synergism for certain metabolites with sleep apnea (G47), prostate hyperplasia (N40), benign neoplasms (D10–D36) and chronic respiratory diseases (J40–J47). Less frequently, dorsalgia (M54), nutritional anemia (D50–D53), polyarthropathies (M05–M14) and gastric/duodenal ulcers (K20–K31) had a synergistic effect with another disease. The metabolites that most frequently benefited from disease synergism were kynurenine, kynurenic acid, Gly and DiMeGly.

Antagonistic effects, where a potential marker loses its potency when the disease of interest co-exists with another diagnosis, were observed too. A marker showing a weak to moderate AUC-ROC for a particular disease often lost some of its significance if applied to a subpopulation with a comorbidity (Table S4). The most frequent comorbidities that decreased the markers’ performance were, unexpectedly, a history of chronic tonsillitis (J35) and varicose veins of the lower extremities (I83). The metabolites that most frequently lost their potential biomarker performance were C5 and C4, acylcarnitines and Leu+Ile. It could be noted that these metabolites are metabolically related, since C5-carnitine is an intermediate in leucine catabolism.

The antagonistic effects, where a potential marker for two separate diseases loses its potential if these two diseases co-exist, were only single cases (Table S5). It should be noted, however, that certain combinations, like anemia with diabetes, were too rare to be considered for statistical analysis. Therefore, the fact that there is no report on their interactions does not mean that there are none.

### 2.7. Pooling of Diagnoses

**Figure 5:**
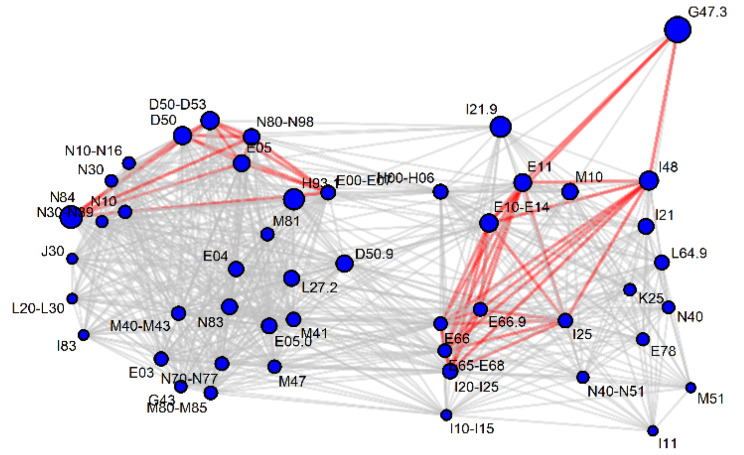
Significance (gray line *p* < 0.05, red line *p* < 0.001) of the AUC-ROC value after the pairwise pooling of diagnosis codes, for which leucine+isoleucine appeared as a potential marker. The size of the vertices is a function of the AUC-ROC value for the diagnosis code, if analyzed independently of the comorbidities. For other metabolites, see Supplementary Figure S1.

Finally, ICD-10 codes with a particular metabolite as a potential marker were pooled, and whether the AUC-ROC remained significantly above 0.5 was tested. The highest AUC-ROC values for combined diagnoses were between 0.7 and 0.8 and pooling more than two or three diagnoses led to a decrease in the AUC-ROC. The results of pairwise pooling were presented as a network of diagnosis codes for a particular metabolic marker candidate (Leu+Ile is given in Figure 5 and other metabolites are given in Figure S1). In most cases, two separate clusters, connected by relatively fewer lines than within the clusters, appeared. For 2-hydroxybutyrate, Ala, C4, C16 and C18 acylcarnitines and other lipid species, the second cluster was minimal in size and for Val, Leu+Ile, Gly, uric acid, betaine and SDMA, the two clusters were roughly equal.

One cluster usually had cardiovascular (I codes), obesity (E66) and diabetes codes (E10–E14), and the other cluster had anemia (D50–D53), hypothyroidism (E00–E07), urogenital tract disorders (N codes) and skin disorders (L codes). One could expect that diseases can increase or decrease the serum concentration of a biomarker (e.g., anemia decreases most amino acids in serum but obesity increases the amino acid levels) and thus two clusters appear. The antagonistic effects reported in the previous chapter suffered from the problem that too few participants had two potentially antagonizing diseases simultaneously. We propose that these networks generated from diagnosis pooling are better indicators of antagonistic diagnosis interactions.

The connecting diseases which maintained a significant AUC-ROC with either cluster were most frequently: hypertension (I10–I15), dorsopathies (M50–M54), ischemic heart diseases (I20–I25) and obesity (E65–E68). Figure 3 demonstrates that hypertension and back pain diagnoses together with chronic upper respiratory track complaints and I30–I52 (other heart diseases, including atrial fibrillation) are common comorbidities for many diseases. Therefore, the relation may remain significant, because the people behind both diagnoses are largely the same individuals.

## 3. Discussion

The search for novel biomarkers for different medical conditions is a very intense field of research. Despite the high number of preliminary reports, only a few make it into clinical practice. The classical approach is to recruit patients with a chosen disease, find matched controls, identify the differences in the level of metabolites, proteins or any other characteristics and then move on to validate these preliminary findings in larger cohorts. In this study, we have attempted an unorthodox design—we picked certain proposed markers and screened them against nearly a complete spectrum of ICD-10 diagnoses. First, this approach may find or confirm markers as the classical validation studies do. Second, it gives a better estimation of the sensitivity and selectivity of the marker for any disease and allows comorbidities that interfere with its performance to be mapped.

The volunteers agreeing to participate in the study are slightly older in terms of the average than the general population. As the number of comorbidities increases with age and the population as a whole ages, this deviance is not a weakness of the study. Nevertheless, studies with even more participants and a better representation of the population should be performed in the future.

For individual metabolites, the calculated AUC-ROCs did not have excellent or very high values. The values of the top performers are, however, in good accordance with previously reported ones for uric acid [24], kynurenine [25] and BCAAs [26]. Their mediocre values do not mean that they cannot be used in practice, since, for instance, uric acid is an important criterion in gout diagnosis despite its AUC-ROC being around 0.7. Additionally, many factors—including comorbidities in patient and control groups, which we sought to study in higher detail—may reduce the performance of potentially useful markers.

The markers chosen for the study were based on a literature search, but in many cases the AUC-ROC values have not been reported previously. For diabetes, hydroxybutanoic acids have been proposed as potential markers [14,21,22], but only 2-hydroxybytyrate was confirmed by our results. Uric acid and homocysteine showed some, although not a strong, relation with cardiovascular diseases and hence are in general agreement with previous publications [13,23,27]. Lyso- and diacyl-phosphatidylcholines or certain other amino acids, beside BCAA, did not turn out to be as promising as suggested earlier through more specific studies [11,14].

PCA is an analytical technique which attempts to group similar patterns in variables into a lesser number of “principal components” and thereby reduce the amount of data and make the highest variations easier to notice. We applied it to the matrix of AUC-ROC values for the metabolite and ICD-10 combinations. The results indicate that certain metabolites are more frequently affected by diseases and separate these diseases from other ICD-10 codes. It is noteworthy that the deviating diseases are related to different organ systems. Hypertension (I10–I15), diabetes (E10–E14; mainly type II in the study population), dyslipidemias (E78) and chronic cardiac ischemia (I25) have been known to be closely related to each other and to metabolic syndrome [28]. Anemias (D50–D53), chronic lung diseases (J40–J47), genital tract disorders (N40–N51; prostate hyperplasia mainly) and senile cataracts (H25), however, appear to share markers with the metabolic syndrome-related diseases. Most people with senile cataracts have hypertension; thus, we cannot tell whether the same metabolites are associated with both diseases or only one. A comparison of Figure 2 and Figure 3 suggests that, in most cases where patients have two overlapping diseases, it does not necessarily mean that the diseases share marker profiles. Therefore, it should not be assumed that a cataract, not to mention chronic respiratory disease etc., co-localize in PCA with metabolic syndrome-related diseases due to an incidental bias in our study group. In fact, meta-analyses have found that hypertension increases the risk of cataracts, and they may indeed have a common biochemical background [29].

The composition of the control group has always been seen as a potential source of bias [30]. Ideally random and completely healthy individuals matched by age and sex are desired, but in practice patients with seemingly unrelated disorders are taken. In our study population, where myopia, dental caries, etc. were also considered diagnoses, no person was without an ICD-10 diagnosis. After removing certain diagnosis codes, which, despite having a unique phenotype, are not always seen as sicknesses, a subpopulation of “completely healthy” subjects was created. The AUC-ROC curves were calculated in comparison to this healthy group as well as the general population. Unexpectedly, the comparison with the healthy group made the markers perform better, with an almost 30% increase in the AUC-ROC values to significantly above 0.5. Considering that the number of healthy controls was smaller than the general population, leading to larger error margins, the increase may sound huge. However, in direct comparison of the results or the application of stricter significance thresholds (not shown), the healthy control group yielded no more than 10–15% more marker candidates. Thus, the results indicate that a sufficiently large and heterogeneous control group is not inferior to well-chosen hand-picked controls. Even more, for possible future clinical application, a heterogenous control group would be the more relevant one [30].

The dependence of the AUC-ROC values on comorbidities was studied further. First, how the performance changes if an underlying comorbidity is present in both the disease and control group was tested. The most interesting are the cases where a metabolite does not have any association with individual diagnosis codes but becomes significant if the two diseases are simultaneously present. The list of markers and disease combinations was long, but due to the limited statistical power it should be taken cautiously. The diagnosis group consisting of migraine, headache and sleep apnea (G40–G47) was one with several markers highly dependent on underlying comorbidities. Interestingly, associations with anemias or myocardial infarction have also been observed in other studies [31,32,33]; this makes one wonder if oxygen metabolism is the connecting point. Heart failure was also associated with kynurenin in the general population, but in subpopulations with other cardiovascular or chronic respiratory diseases, its AUC-ROC increased to values that could be usable in practice with minimal supporting criteria.

The metabolites that were markers for both the disease of interest and the underlying comorbidity rarely lost their association with those diseases. For instance, hypertension (I10–I15) and cardiac ischemia shared (I20–I25) C4 and C5 carnitine esters and urea as potential markers when compared to the general population. The significance was lost if the control group had the other diagnosis present. The same happened with Orn, Glu and urea for cardiovascular diagnoses (I codes) in obese/dyslipidemic (E66/E78) populations.

Next, the synergistic and antagonistic effects in co-existing diagnoses were studied. Once again, a loss of association (antagonism) was rarely observed, but as pointed out earlier there may have been too few participants with “antagonistic” diseases simultaneously present. Synergism was most apparent between hypertension (I10–I15) and obesity (E66), with the metabolism of amino acids and their derivates (kynurenine, kynurenic acid, DiMeGly) being much more out of place than obesity or hypertension alone. It is known that obesity may cause hypertension and both diagnoses are partially regulated by the same hormonal mediators [34,35]. On the metabolome level, the interactions are less clear and we are limited to comparing obesity metabolomics with cardiovascular metabolomics [36]. While discussing one’s results, it is common practice to highlight which markers are shared between possibly linked diseases. Markers for disease synergism have so far been scarce.

The final set of analyses consisted of pooling diagnoses and comparing the performance against healthy controls. Diagnosis codes are artificial constructs based on our knowledge and convenience in classifying medical problems. Pathological mechanisms and principles are shared by many diseases. Metabolic biomarkers might better describe molecular processes than ICD-10 codes. A good example from the world of proteins is C-reactive protein, which is among the most used biomarkers although it only reveals acute phase reaction and not a specific diagnosis. Pooling diseases with a common pathology may therefore reveal markers that underperform under usual classification constraints.

Within the limits of confidence intervals, pooling did not increase the performance compared to the best individual diagnoses taken for pooling. A decrease in performance was more common and is illustrated with two, not one, clusters in the network analysis (Figure 5 and Figure S1). The explanation is simply the increase/decrease in the direction of the metabolite concentration depending on the health status. For some metabolites (e.g., long-chain acylcarnitines), only unidirectional changes were seen, and pooling patients with different diseases therefore did not drastically decrease the marker performance.

This study also has several shortcomings, which should be kept in mind when interpreting the results. First, the participants are all on treatments according to their needs. The severity, medications and success in the treatment control have not been taken into account. Well-controlled chronic diseases likely show less metabolic disturbances than those with more severe conditions that are harder to control. Additionally, in certain cases the observed changes in metabolites may stem from the treatment and not the disease itself. Hence, these biomarkers and their relations are more relevant for estimating the performance of monitoring or prognostic biomarkers and not for early detection biomarkers.

Second, this report includes only the most robust analysis with no adjustment for age, gender and other potential covariates. While corrections can also be made in bedside medicine, markers requiring less manipulations are preferred. In this report, the aim was to provide a robust foundation for the systematic mapping of disease and metabolite associations and to characterize the role of comorbidities. In future studies, more complex layers of details and adjustments with additional covariates can and should be attempted.

## 4. Materials and Methods

### 4.1. Subjects Recruitment and Clinical Data Collection

Subjects partaking in the current study were selected from individuals who had already donated their blood samples to the Estonian Genome Project for genetical analysis. Alongside blood sample donations, participants filled in a thorough questionnaire, including diagnoses throughout their lives. The self-reported data have been further supplemented and corrected with the available medical records, which are expected to comprehend most diagnostic events in Estonia in at least the last two decades. For the current study, the patient age and reported diseases were extracted from the general database, and no additional questionnaires were used. The study was approved by the Research Ethics Committee of the University of Tartu (permission 264/T-5).

For serum samples, re-recruitment letters were sent to 2486 adult individuals. The response rate was 43%. The serum samples from volunteers were collected in 5 mL Vacutainer tubes containing silicone and micronized silica particles to accelerate the clotting process. The samples were left to clot for 1 h at room temperature and centrifuged at 1300× *g* for 20 min. The obtained serum samples were aliquoted and stored at −80 °C until analysis. Unfortunately, the documentation on whether the serum was taken after proper fasting is incomplete, and the samples should therefore be treated as random time samples. However, PCA and histogram analyses of targeted analysis or untargeted profiles did not reveal any outliers or suspicious subgroups (data not shown).

### 4.2. Sample Preparation and Analysis

For quality control pooled serum from all the study participants was used. Each batch consisted of a blank (water + internal standard), quality control sample and 18 random samples from the study group.

For amino acid and acylcarnitine measurements, 20 μL of serum was mixed with 100 μL of internal standards (supplementary Table S5) and 750 μL of ice-cold methanol. After 10 min at −20 °C, proteins and salts were removed by centrifugation at 21,000 *g* for 10 min. The supernatant was dried under a stream of nitrogen for 3 h at room temperature. The dry residue was derivatized with 60 μL of 3 M HCl/butanol at 65 °C for 15 min, dried again under a nitrogen stream and dissolved in 100 μL of methanol with 0.2% formic acid. Twenty microliters were injected into a mass spectrometer (Sciex Qtrap 3200, with Shimadzu Prominence liquid chromatography). A direct infusion of the sample was conducted with 30% water and 0.2% formic acid in acetonitrile as the carrier eluent. Acylcarnitines were analyzed as precursors of *m*/*z* = 85 fragments, and phosphatidylcholines as precursors of *m*/*z* = 184. Amino acids were analyzed by a multiple reaction monitoring (MRM) scan (details in Supplementary Table S5).

All the other compounds were measured with no derivatization by mixing 50 μL of serum with 10 μL of internal standards, followed by protein precipitation as described above. The analyses were conducted using Agilent 1260 series liquid chromatography coupled with MS Sciex Qtrap 4500 mass spectrometry. A C18 4 mm × 2 mm, 10 µm precolumn (Phenomenex, Torrance, CA, USA) was used for chromatographic separation. The gradient was composed of methanol and water, both with 0.2% formic acid. The gradient started with 5% methanol for 1 min, followed by a linear increase to 95% methanol within 5 min and, finally, washing with 95% methanol for 4 min. The MRM transitions and ionization parameters are given in Supplementary Table S5.

### 4.3. Statistics

The liquid chromatography-mass spectrometry peak integration was conducted automatically by the Analyst program, but it was always confirmed by manual inspection, and for low-abundance metabolites, the manual correction of peak identification was frequently necessary. The obtained raw data were transferred to Microsoft Excel, which was used to visualize and correct batch effects, as well as to generate input files for further statistical analysis in R 3.6.1. First, all the metabolite signals of interest were normalized to their respective internal standard (Supplementary Table S5) in the same sample. Missing data were <2% of the whole data and were substituted by the batch average for their respective metabolite. Second, the blank was subtracted as the background and all the data were normalized to the batch average for their respective metabolite. The coefficient of variation in the quality control was 16% on average, but exceptionally high for eicosapentanenoic and arachidonic acids (59% and 57% respectively).

The AUC-ROC calculation and related statistics were conducted using the pROC package [37]. The networks were built with the Network package [38]. The error margins presented throughout the article are standard deviation, if not stated otherwise.

## 5. Conclusions

In conclusion, we explored a list of serum metabolites and how well they would perform as markers for ICD-10 codes. In individual cases, the metabolites reached moderate AUC-ROC values, but neither alone nor in combination with comorbidity data were they sufficient for clinically desired sensitivities and specificities. This does not mean that they could not be used in combination with other investigations and clinical data. Coexisting diseases may cause drastic changes in the metabolites’ sensitivity and specificity for a given disease.

## Figures and Tables

**Table 1:** 10th revision of the international classification of diseases (ICD-10) codes and the groups with the highest prevalence in the study population characterized by their mean age, age limits and gender proportions.

ICD-10 Code	Cases	Mean Age (Min–Max) (Years)	Male%
J30–J39; diseases of upper respiratory tract	395	51.5 (23–86)	50%
I10–I15; Hypertension	353	64.1 (28–89)	58%
M50–M54; Other dorsopathies	347	58.5 (22–87)	57%
I30–I52; Other forms of heart disease	325	61.2 (23–89)	53%
J35; Chronic disease of tonsils and adenoids	272	51.0 (23–86)	43%
M51; Other intervertebral disk disorders	220	59.3 (25–87)	60%
I11; Hypertensive heart disease	197	66.6 (36–89)	57%
I49; Atrial fibrillation	192	62.2 (23–89)	56%
K20–K31; Diseases of stomach and duodenum	192	59.7 (25–87)	60%
D10–D36; Benign neoplasms	191	59.1 (24–81)	31%
I80–I89; Disorders of veins and lymph system	186	60.0 (26–87)	39%
G40–G47; Episodic and paroxysmal disorders	185	56.9 (26–86)	39%
I10; Primary hypertension	164	61.3 (28–82)	59%
M15–M19; Arthrosis	164	64.6 (31–87)	43%
E00–E07; Disorders of thyroid gland	157	58.7 (22–89)	15%
I83; Varicose veins of lower extremities	150	61.1 (26–87)	35%
E70–E90; Metabolic disorders	141	59.6 (25–86)	46%
E66; Obesity	135	58.1 (25–83)	45%
E65–E68; Obesity and other hyperalimentation	135	58.1 (25–83)	45%
M20–M25; Other joint disorders	135	57.9 (23–86)	49%
L60–L75; disorders of skin appendages	129	50.0 (23–90)	68%
H90–H95; Other diseases of ear	128	65.2 (31–89)	63%
N40–N51; Diseases of male genitals	128	65.7 (26–90)	100%

**Table 2:** Highest AUC-ROC values for metabolite and ICD-10 code combinations. The full list is in Supplementary Table S1.

ICD-10 Diagnose	Marker	Cases	AUC-ROC (CI 95%)	Regression Beta	Beta *p*-Value
E10–E14; Diabetes	Ala	63	0.72 (0.66–0.78)	2.2 × 10^−2^	1.0 × 10^−9^
I50; Heart failure	Kyn	90	0.72 (0.66–0.77)	4.1 × 10^2^	8.1 × 10^−10^
E11; Type II diabetes	Val	55	0.7 (0.63–0.76)	2.7 × 10^−2^	2.0 × 10^−6^
E11; Type II diabetes	C3	55	0.69 (0.62–0.76)	1.8	3.1 × 10^−4^
M10; Gout	Uric acid	26	0.69 (0.57–0.81)	4.7 × 10^2^	4.9 × 10^−5^
E10–E14; Diabetes	Leu+Ile	63	0.67 (0.6–0.75)	1.9 × 10^−2^	8.8 × 10^−6^
D50–D53; Nutritional anemias	Leu+Ile	55	0.67 (0.61–0.74)	−3.1 × 10^−2^	5.8 × 10^−5^
I50; Heart failure	Urea	90	0.67 (0.61–0.72)	4.1	1.0 × 10^−6^
I25; Chronic cardiac ischemia	Kyn	75	0.66 (0.6–0.73)	3.2 × 10^2^	2.9 × 10^−6^
H25; Senile cataract	ADMA	85	0.66 (0.6–0.72)	3.1 × 10^3^	4.5 × 10^−5^
E11; Type II diabetes	DiMeGly	55	0.66 (0.59–0.73)	1.9 × 10^2^	1.6 × 10^−4^
D50–D53; Nutritional anemias	Uric acid	55	0.66 (0.58–0.73)	−3.0 × 10^2^	7.2 × 10^−5^
I25; Chronic cardiac ischemia	Urea	75	0.66 (0.6–0.72)	4.1	4.4 × 10^−6^
I20–I25; Ischemic heart diseases	DiMeGly	107	0.66 (0.61–0.71)	1.7 × 10^2^	6.4 × 10^−6^
N80–N98; Noninflammatory disorders of female genital tract	Betaine	74	0.66 (0.59–0.72)	−2.5 × 10	1.0 × 10^−5^
N80–N98; Noninflammatory disorders of female genital tract	Uric acid	74	0.65 (0.59–0.72)	−3.4 × 10^2^	6.6 × 10^−7^
E05; Hyperthyroidism	Leu	66	0.65 (0.58–0.72)	−2.4 × 10^−2^	2.5 × 10^−4^
E10–E14; Diabetes	Glu	63	0.65 (0.58–0.72)	4.0 × 10^−2^	9.3 × 10^−5^
D50–D53; nutritional anemias	Glu	55	0.65 (0.57–0.72)	−5.6 × 10^−2^	4.6 × 10^−4^
I50; Heart failure	Kyn. acid	90	0.65 (0.59–0.71)	2.8 × 10^3^	1.3 × 10^−7^
N40; Hyperplasia of prostate	DiMeGly	105	0.65 (0.59–0.7)	1.8 × 10^2^	2.1 × 10^−6^
N80–N98; Noninflammatory disorders of female genital tract	Leu	74	0.65 (0.59–0.71)	−2.1 × 10^−2^	7.9 × 10^−4^
I11; Hypertensive heart disease	Urea	197	0.64 (0.6–0.69)	4.2	1.4 × 10^−9^
E05; Hyperthyroidism	Val	66	0.64 (0.58–0.71)	−2.1 × 10^−2^	2.7 × 10^−4^
M13; Other inflammatory arthritis	lysoPC (C22:6)	63	0.64 (0.58–0.71)	1.5	4.9 × 10^−4^
D50–D53; Nutritional anemias	Urea	55	0.64 (0.57–0.72)	−5.4	3.5 × 10^−4^

**Table 3:** Top AUC-ROC values for the simultaneous presence of two diagnoses where one of them (underlying disease) is also present in the control group. For comparison, the AUC-ROC values for both individual diagnoses are given.

Metabolite	Disease of Interest			Underlying Disease		Overlap	
	ICD-10	Cases	AUC-ROC	ICD-10	AUC-ROC	Cases	AUC-ROC
C5	I20–I25	107	0.63 (0.57–0.68)	E00–E07	0.59 (0.55–0.64)	17	0.82 (0.73–0.9)
C4	I10–I15	353	0.59 (0.55–0.62)	H91.9	0.48 (0.41–0.56)	31	0.81 (0.7–0.92)
Kynurenine	I50	22	0.81 (0.71–0.92)	I10–I15	0.6 (0.56–0.64)	15	0.82 (0.74–0.91)
Kyn acid ^a^	I50	22	0.75 (0.63–0.88)	I10–I15	0.59 (0.55–0.63)	15	0.80 (0.7–0.91)
Arg	D10–D36	191	0.56 (0.51–0.6)	I25	0.56 (0.5–0.63)	16	0.80 (0.67–0.94)
lysoPC(C18:0)	G40–G47	185	0.53 (0.48–0.58)	I40	0.49 (0.41–0.58)	15	0.81 (0.69–0.93)
SDMA	G40–G47	185	0.55 (0.51–0.6)	I40	0.57 (0.49–0.65)	15	0.84 (0.72–0.95)
Val	E66	112	0.63 (0.57–0.68)	I49.9	0.51 (0.47–0.56)	25	0.80 (0.73–0.87)
lysoPC(C16:0)	M05–M14	116	0.58 (0.52–0.64)	I50	0.56 (0.5–0.62)	16	0.83 (0.71–0.95)
Hexoses	J40–J47	81	0.64 (0.57–0.7)	I80–I89	0.50 (0.46–0.55)	17	0.81 (0.72–0.89)
Kynurenine	I50	90	0.72 (0.66–0.77)	J30	0.53 (0.47–0.59)	16	0.82 (0.74–0.91)
Kynurenine	I20–I25	107	0.65 (0.59–0.7)	J30–J39	0.51 (0.47–0.55)	32	0.80 (0.74–0.87)
Kynurenine	I50	90	0.72 (0.66–0.77)	N40	0.60 (0.54–0.66)	16	0.84 (0.73–0.96)
Gly	G40–G47	185	0.50 (0.46–0.55)	D50–D53	0.51(0.43–0.6)	17	0.81 (0.69–0.93)

^a^ Kyn acid—kynurenic acid.

**Table 4:** ICD-10 code combinations that have a significant synergistic or antagonistic effect on the AUC-ROC value. The table is restricted to combinations with cases >24. Complete tables with *p* values are provided in Tables S3 and S4 for the synergistic and antagonistic effects, respectively.

Metabolite	Disease 1		Disease 2		Co-Present	
	ICD-10	AUC-ROC	ICD-10	AUC-ROC	Cases	AUC-ROC
Kynurenine	I20–I25	0.65 (0.59–0.70)	J30–J39	0.51 (0.47–0.55)	32	0.79 (0.73–0.85)
Val	E66	0.63 (0.58–0.68)	I49	0.51 (0.47–0.56)	29	0.75 (0.67–0.83)
Kynurenine	E66	0.61 (0.56–0.66)	I11	0.63 (0.59–0.68)	39	0.75 (0.68–0.83)
Uric acid	E66	0.61 (0.56–0.66)	I11	0.63 (0.58–0.67)	39	0.75 (0.68–0.82)
Lactate	J40–J47	0.62 (0.55–0.68)	K20–K31	0.52 (0.48–0.57)	29	0.73 (0.65–0.82)
Val	E66.9	0.63 (0.57–0.68)	I10	0.55 (0.50–0.60)	37	0.72 (0.64–0.80)
Urea	E66	0.57 (0.51–0.62)	I49	0.55 (0.50–0.59)	29	0.72 (0.63–0.82)
Kynurenic acid	E66	0.62 (0.56–0.67)	I11	0.59 (0.54–0.63)	39	0.72 (0.64–0.81)
PC(C40:5)	D10–D36	0.56 (0.52–0.61)	E70–E90	0.61 (0.55–0.66)	29	0.72 (0.64–0.80)
DiMeGly	E66	0.58 (0.53–0.63)	I11	0.59 (0.55–0.63)	39	0.72 (0.65–0.78)
Kynurenine	E66.9	0.61 (0.56–0.66)	I80–I89	0.53 (0.48–0.58)	25	0.72 (0.63–0.80)
Tyr	I49.9	0.61 (0.55–0.67)	J30–J39	0.52 (0.48–0.55)	30	0.72 (0.64–0.79)
Ala	E66	0.61 (0.56–0.66)	I10	0.59 (0.54–0.64)	40	0.71 (0.63–0.79)
Tyr	E66.9	0.58 (0.53–0.63)	I49	0.53 (0.49–0.58)	25	0.71 (0.62–0.79)
Gly	E66.9	0.55 (0.49–0.61)	I11	0.56 (0.51–0.60)	30	0.71 (0.62–0.80)
Gly	M51	0.60 (0.56–0.64)	N40	0.60 (0.54–0.65)	37	0.71 (0.63–0.78)
C3	E66.9	0.59 (0.54–0.65)	I49	0.51 (0.46–0.55)	25	0.71 (0.62–0.79)
2-OH butyrate	I11	0.57 (0.53–0.62)	M05–M14	0.59 (0.53–0.64)	27	0.70 (0.60–0.80)
PC(C38:6)	H91	0.57 (0.51–0.63)	K20–K31	0.55 (0.50–0.59)	30	0.70 (0.60–0.79)
lysoPC(C18:0)	D10–D36	0.58 (0.54–0.62)	E70–E90	0.56 (0.51–0.61)	29	0.70 (0.61–0.78)
Leu	E66	0.60 (0.55–0.65)	I10	0.54 (0.50–0.59)	40	0.70 (0.62–0.77)
PC(C32:1)	M15–M19	0.51 (0.46–0.56)	N40	0.59 (0.53–0.65)	34	0.70 (0.61–0.78)
C2	D10–D36	0.54 (0.49–0.58)	I10	0.60 (0.55–0.64)	38	0.69 (0.61–0.77)
Gly	E66	0.53 (0.48–0.59)	K20–K31	0.54 (0.49–0.58)	34	0.69 (0.58–0.79)
Gly	E10–E14	0.56 (0.48–0.64)	I11	0.56 (0.51–0.60)	31	0.69 (0.60–0.78)
Leu	H90–H95	0.53 (0.47–0.58)	I49	0.53 (0.48–0.58)	31	0.69 (0.60–0.77)
Lactate	I10–I15	0.57 (0.53–0.61)	M05–M14	0.57 (0.51–0.63)	52	0.68 (0.61–0.76)
C0	J40–J47	0.56 (0.5–0.63)	M50–M54	0.54 (0.50–0.58)	37	0.67 (0.59–0.75)
lysoPC(C22:6)	I11	0.57 (0.53–0.62)	K20–K31	0.57 (0.52–0.62)	47	0.67 (0.59–0.74)
Arg	D10–D36	0.56 (0.51–0.60)	M50–M54	0.5 (0.47–0.54)	75	0.64 (0.58–0.70)
Uric acid	I10–I15	0.61 (0.57–0.64)	D10–D36	0.56 (0.51–0.60)	87	0.48 (0.41–0.54)
C5	E00–E07	0.59 (0.55–0.64)	I10–I15	0.60 (0.56–0.64)	64	0.50 (0.44–0.57)
C5	I10–I15	0.60 (0.56–0.64)	N10–N16	0.59 (0.53–0.65)	31	0.48 (0.38–0.58)

## Supplementary Materials

The following are available online at https://www.mdpi.com/2218-1989/10/5/196/s1: Figure S1: biomarker candidate resistance to disease pooling, Table S1: AUC-ROC values for ICD-10 and metabolite pairs, Table S2: AUC-ROC values in the case of an underlying disease, Table S3: synergism between diseases, Table S4: antagonism between diseases, Table S5: ion pairs and liquid chromatography-mass spectrometry conditions for measured metabolites and internal standards.
