# Supplementary material for: Mapping Metabolite and ICD-10 Associations
Source: Metabolites. 2020 May 14;10(5):196. doi: 10.3390/metabo10050196 (PMC7281140; doi:10.3390/metabo10050196)
Supplement: Supplementary file 1 [file metabolites-10-00196-s001.zip › metabolites-789306-pr-sp/Supplementary_Figure_S1.pdf]

Supplementary Figure S1. Biomarker candidate resistance to disease pooling.

Significance (gray line p<0.05, red line p<0.001) of AUC-ROC value after pairwise pooling of diagnose codes for which the specified metabolite appeared as a potential marker. The size of vertexes depends on the AUC-ROC value for the diagnose code if analyzed independently of comorbidities.

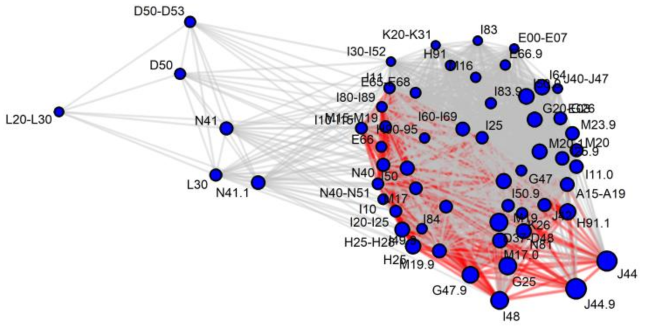

ADMA

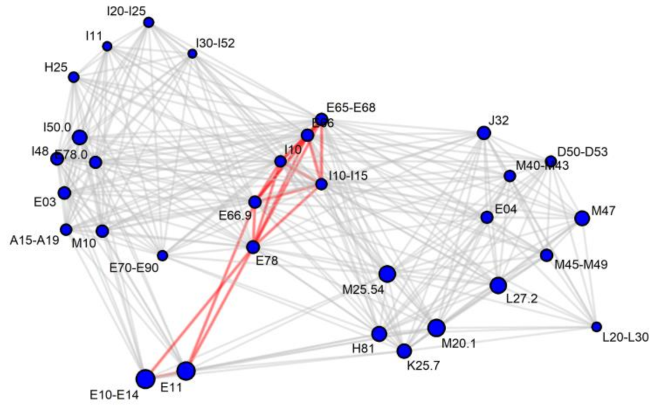

Ala

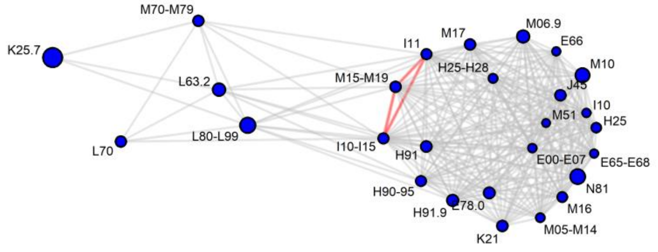

Arachidonic acid

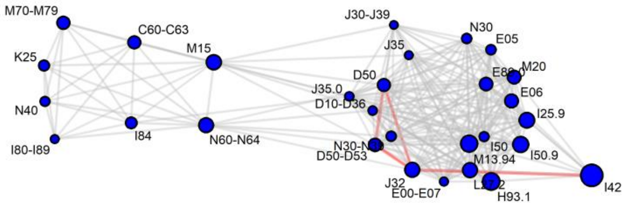

Arg

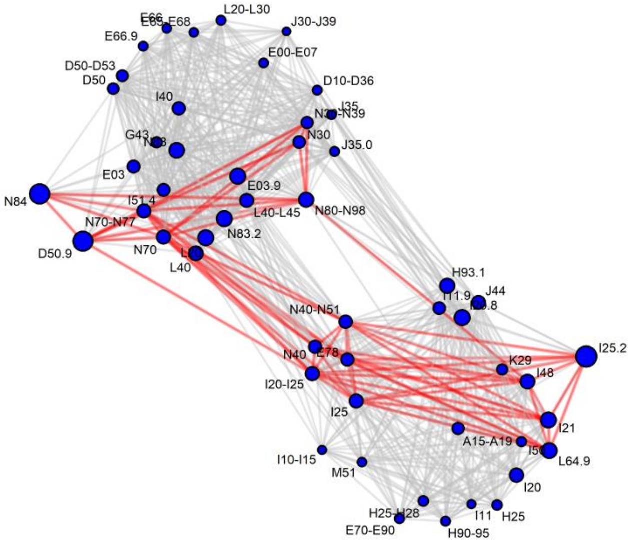

Betaine

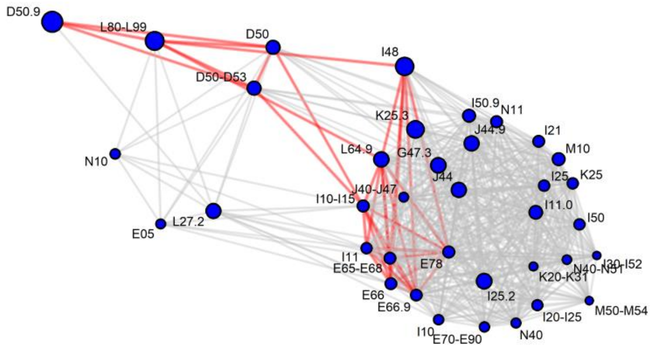

Free carnitine

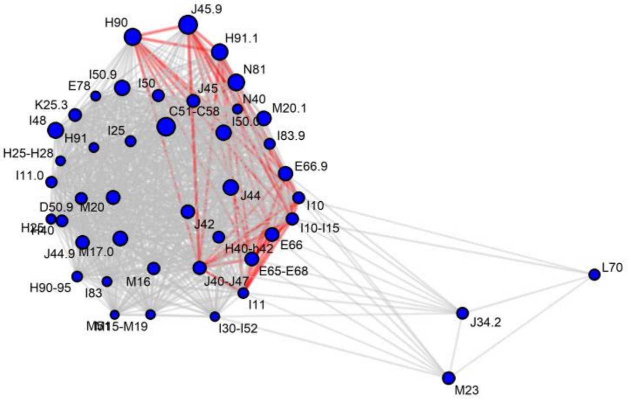

C2 carnitine

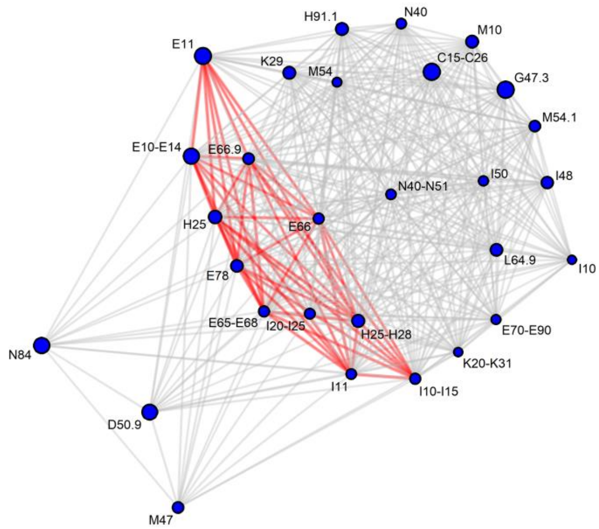

C3 carnitine

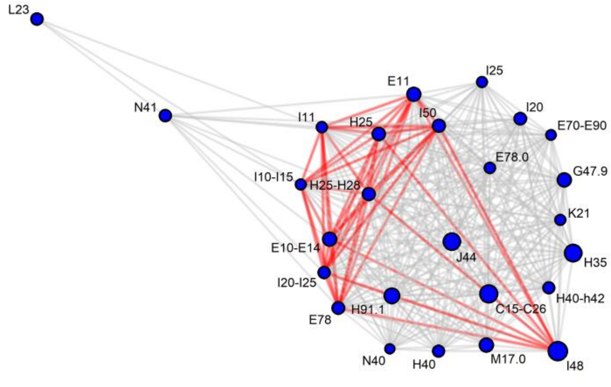

C4 carnitine

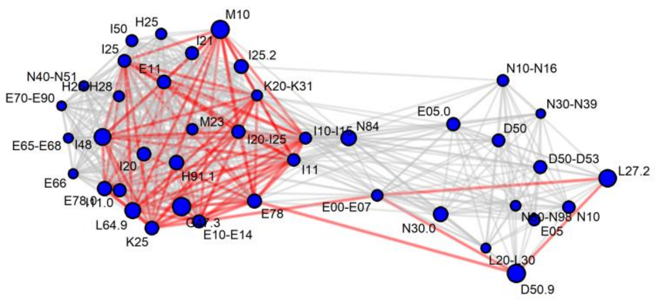

C5 carnitine

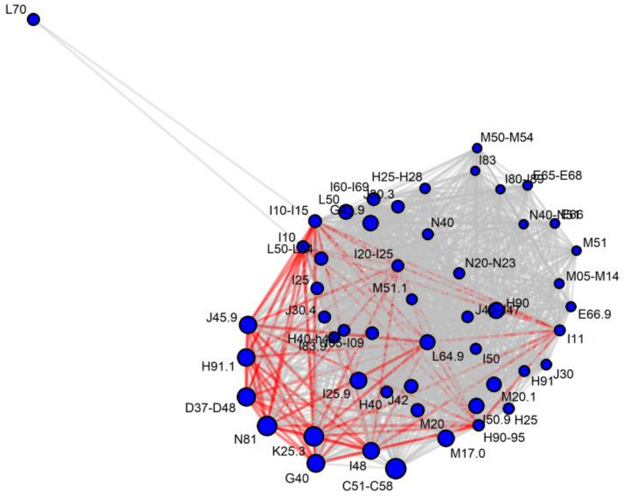

C16 carnitine

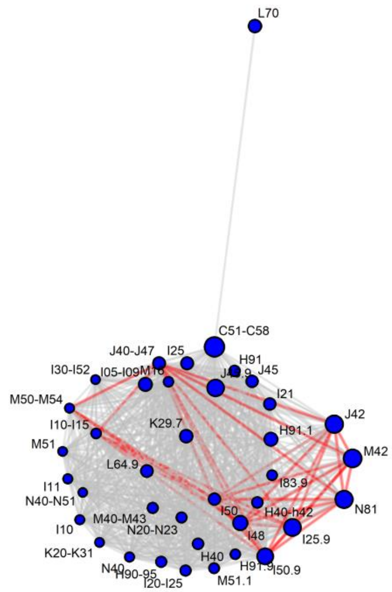

C18 carnitine

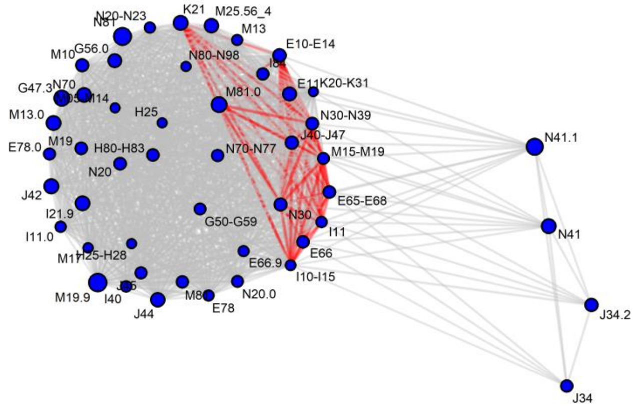

Creatine

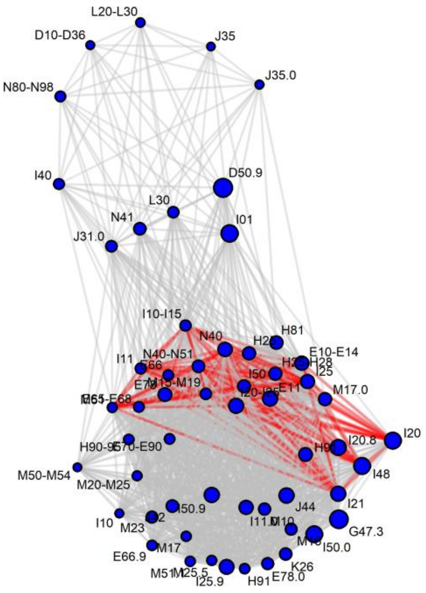

DiMeGly

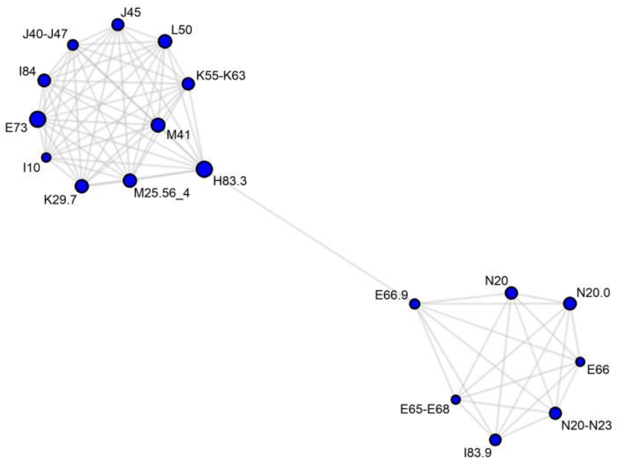

Eicosapentanoic acid

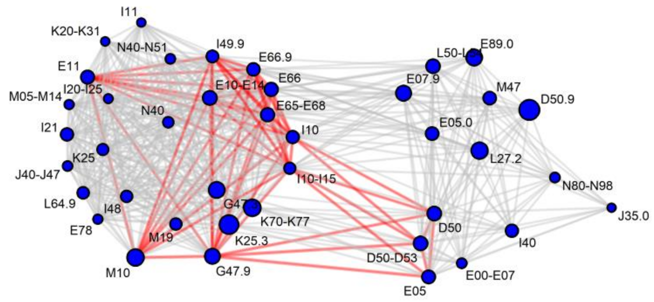
I11
K20-K31
N40-N51
I49.9
E66.9
E11
E66
I20-I25
E10-E14
M05-M14
E65-E68
N40
I21
I10
K25
J40-J47
I10-I15
L64.9
I48
G47
K70-K77
E78
M19
K25.3
M10
G47.9
E89.0
E07.9
M47
D50.9
E05.0
L27.2
N80-N98
J35.0
D50
I40
D50-D53
E00-E07
E05

Glu

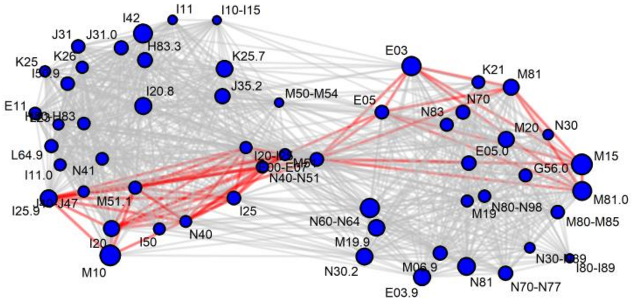
I42
I11
I10-I15
J31
J31.0
H83.3
K26
K25.7
K25
J35.2
I20.8
M50-M54
E11
L64.9
N41
I11.0
M51.1
I25.9
I25
I20
I50
N40
M10
N40-N51
E03
K21
M81
E05
N83
N70
M20
N30
E05.0
M15
G56.0
M81.0
M19
N80-N98
N60-N64
M80-M85
M19.9
N30.2
M06.9
N81
N70-N77
I80-I89
E03.9

Gly

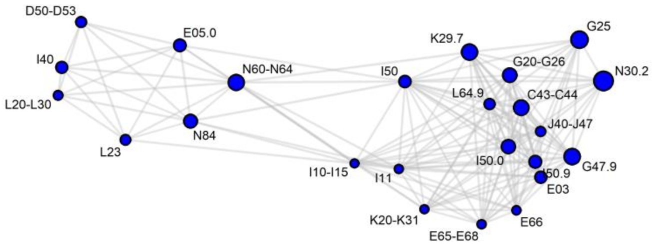
D50-D53
E05.0
I40
N60-N64
L20-L30
L23
N84
K29.7
G25
I50
G20-G26
N30.2
L64.9
C43-C44
J40-J47
I50.0
G47.9
I10-I15
I11
E03
K20-K31
E65-E68
E66

HCys

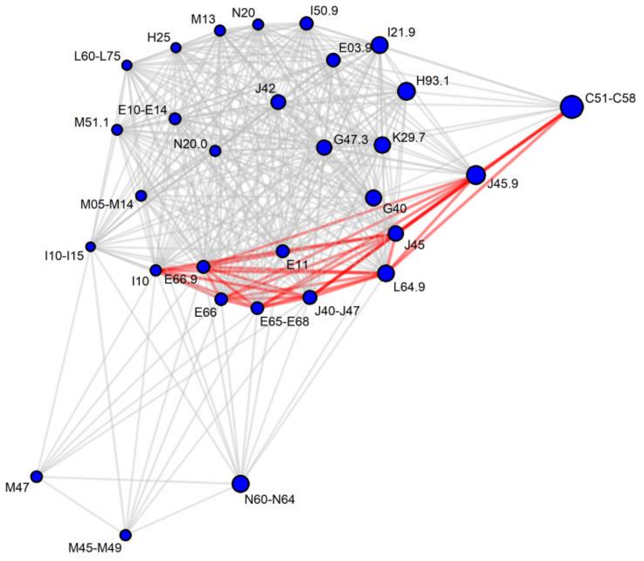

Hexoses

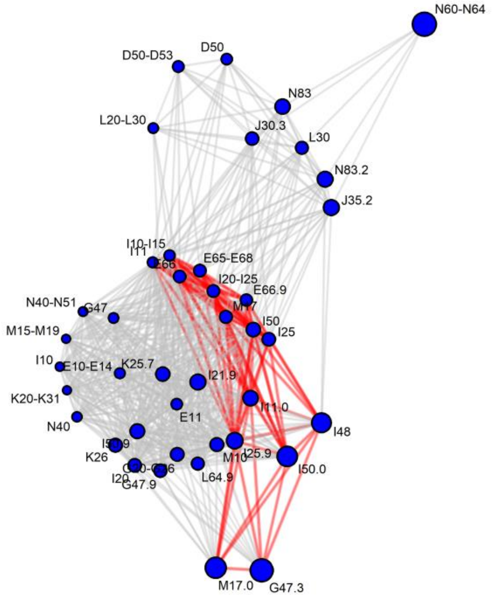

Kynurenic acid

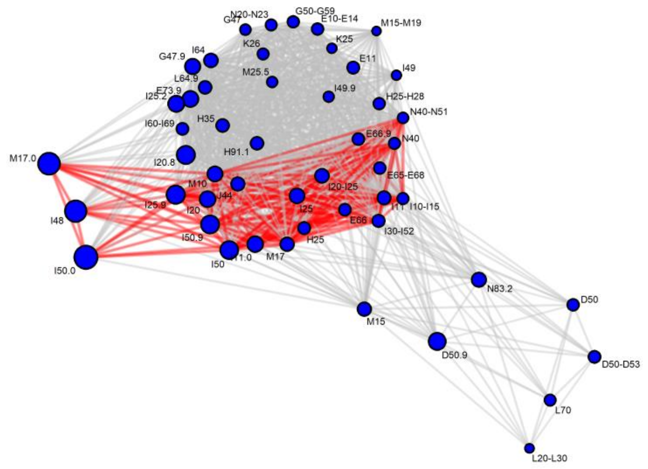

Kynurenin

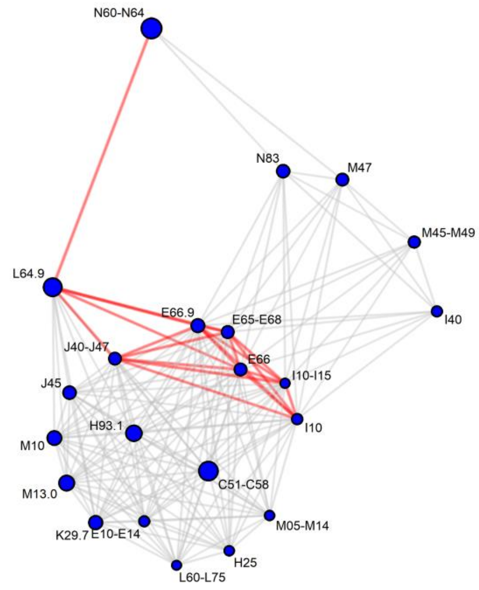

Lactate

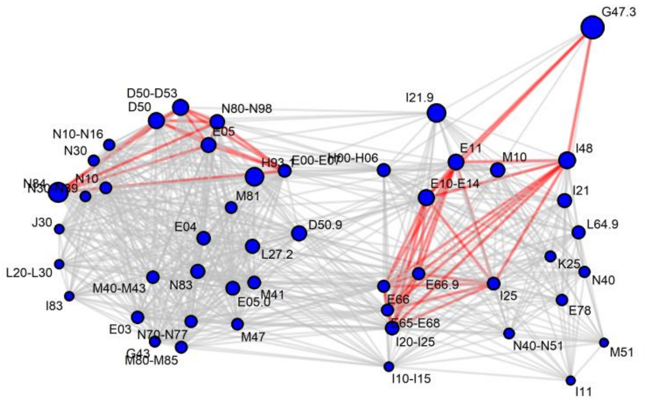
G47.3
I21.9
D50-D53
D50
N80-N98
N10-N16
E05
N30
H93
E00-E07
H00-H06
E11
M10
I48
N10
E10-E14
I21
M81
J30
D50.9
L64.9
E04
L27.2
K25
L20-L30
M40-M43
N83
E66.9
N40
I83
E05.0
M41
E66
I25
E78
E03
N70-N77
M47
E65-E68
I20-I25
N40-N51
M51
M80-M85
I10-I15
I11

Leu+Ile

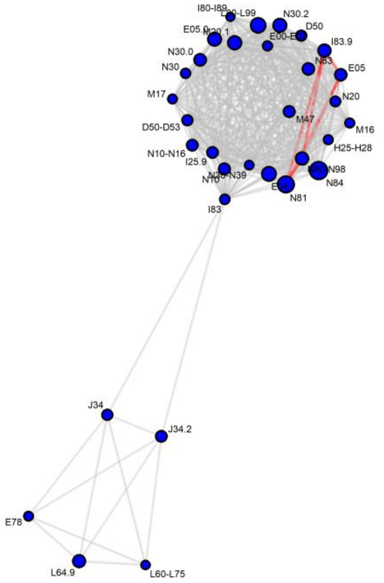
N30.2
D50
I83.9
N30.0
N83
E05
N30
N20
M17
M47
M16
D50-D53
H25-H28
N10-N16
I25.9
N98
N84
N81
I83
J34
J34.2
E78
L64.9
L60-L75

Met

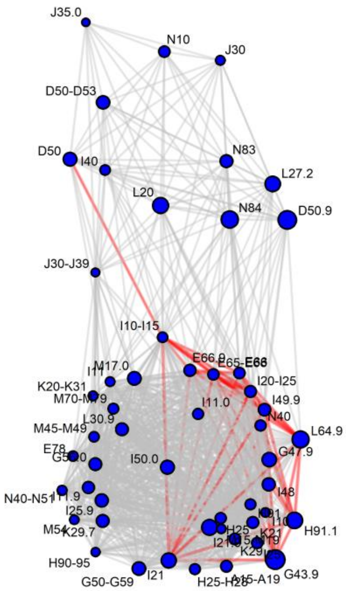
J35.0
N10
J30
D50-D53
D50
I40
N83
L27.2
L20
N84
D50.9
J30-J39
I10-I15
E66.9
I20-I25
M17.0
K20-K31
M70-M79
I11.0
I49.9
N40
M45-M49
L30.9
L64.9
E78
G47.9
I50.0
N40-N51
I48
I25.9
M54
K29.7
H91.1
H90-95
G50-G59
I21
H25-H28
A15-A19
G43.9

Orn

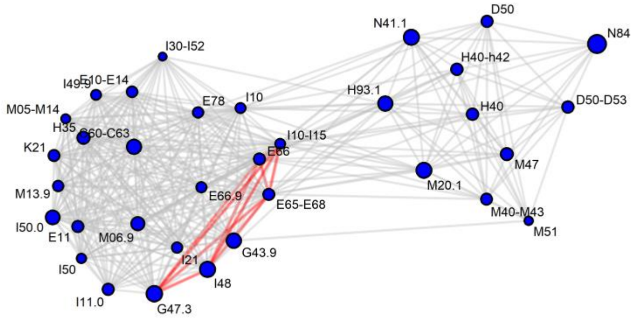
N41.1
D50
N84
I30-I52
H40-h42
I49.9
E10-E14
H93.1
D50-D53
M05-M14
E78
I10
H40
H35
C60-C63
I10-I15
K21
E66
M47
M20.1
M13.9
E66.9
E65-E68
M40-M43
I50.0
M51
E11
M06.9
G43.9
I21
I50
I48
I11.0
G47.3

Phe

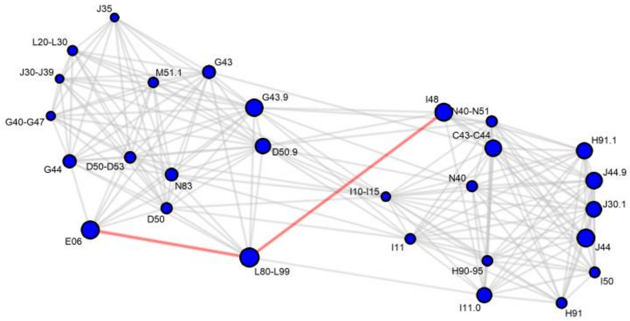
J35
L20-L30
G43
J30-J39
M51.1
G43.9
I48
N40-N51
G40-G47
C43-C44
H91.1
D50.9
G44
D50-D53
J44.9
N40
N83
I10-I15
J30.1
D50
E06
I11
J44
L80-L99
H90-95
I50
I11.0
H91

SDMA

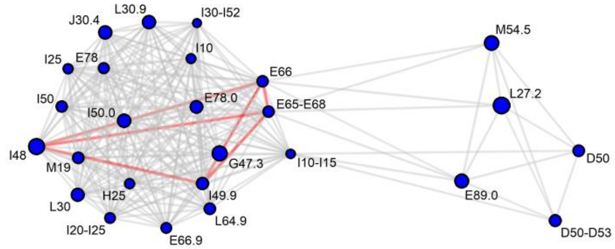

Tyr

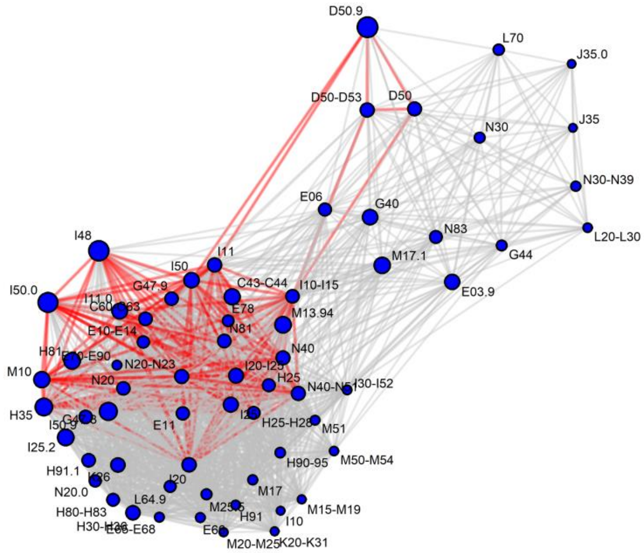

Urea

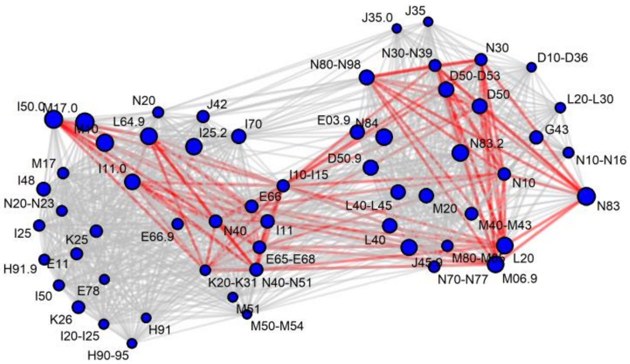

Uric acid

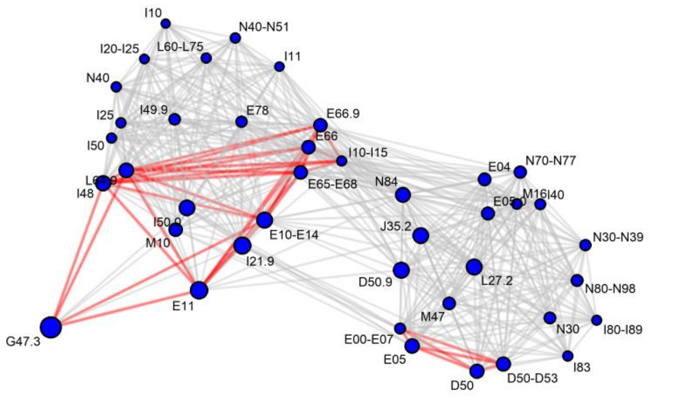

Val

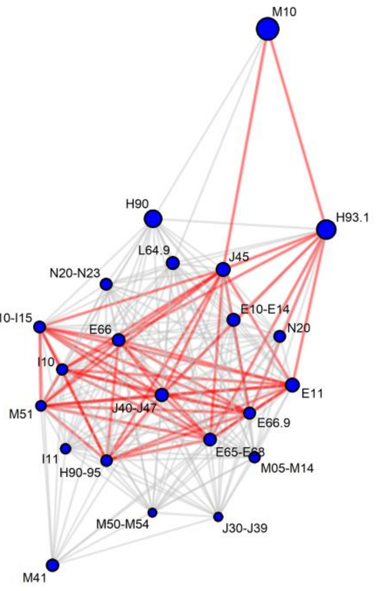

2-hydroxybutyrate

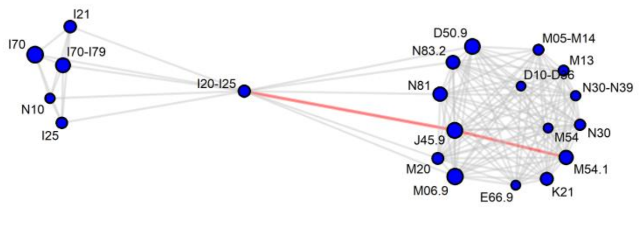

LysoPC(C16:0)

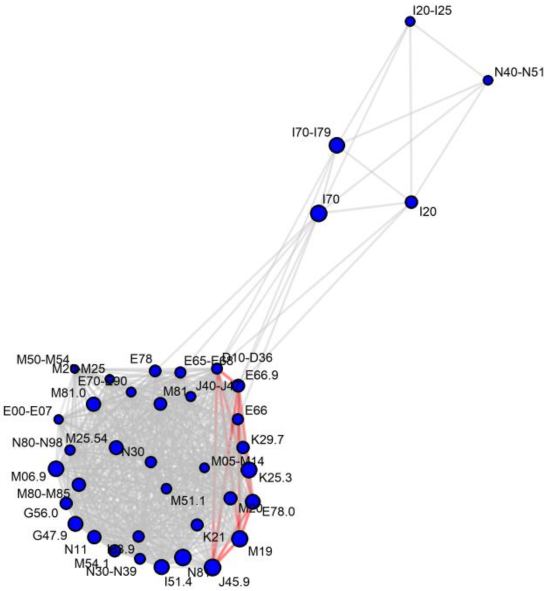

LysoPC(C18:0)

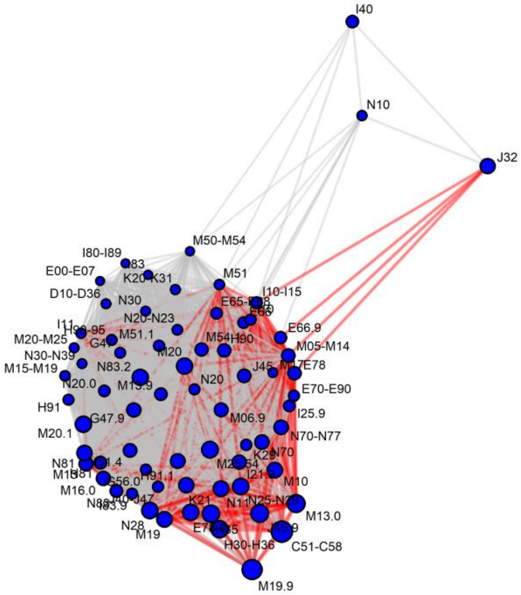

LysoPC(C22:6)

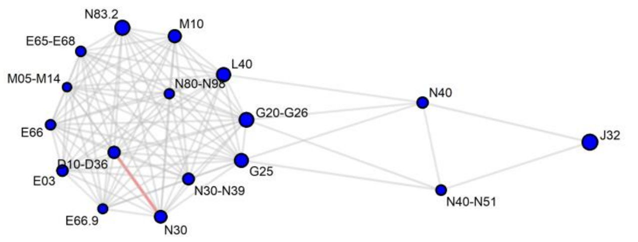

PC(C32:1)

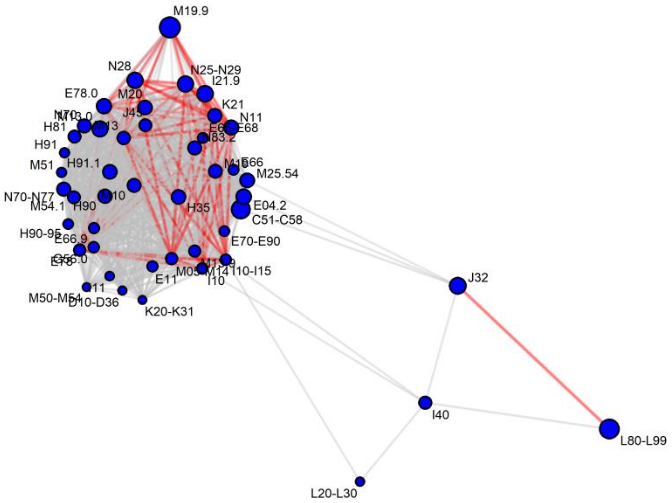

PC(C38:6)

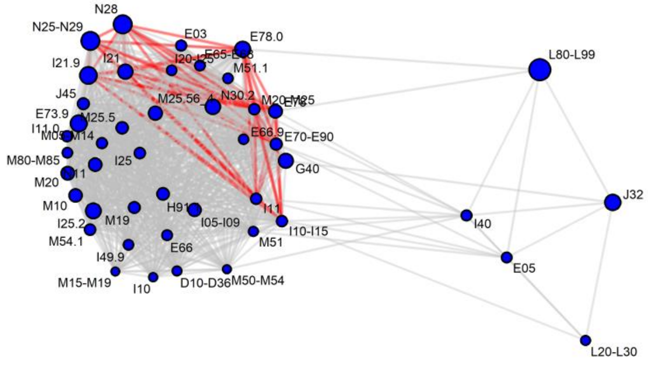

PC(C40:5)
